# Swedish version of measuring cultural awareness in nursing students: validity and reliability test

**DOI:** 10.1186/s12912-016-0146-6

**Published:** 2016-04-14

**Authors:** Emina Hadziabdic, Jalal Safipour, Margareta Bachrach-Lindström, Sally Hultsjö

**Affiliations:** Department of Health and Caring Science, Faculty of Health and Life Sciences, Linnaeus University, 351 95 Växjö, Sweden; Department of Medical and Health Sciences, Linköping University, 581 83 Linköping, Sweden; Department of Nursing, School of Health Sciences, Box 1026, 551 11 Jönköping, Sweden; Psychiatric Clinic, County Hospital Ryhov, 55185 Jönköping, Sweden

**Keywords:** Cultural competency/education, Cultural diversity, Factor analysis, Statistical, Health, Knowledge, Attitudes, Practice, Psychometrics, Students, Nursing, Surveys and questionnaires/standards, Transcultural nursing

## Abstract

**Background:**

Nearly 20 % of the Swedish population is foreign-born. Increased exposure of patients from diverse cultures means there is an urgent need to address their unique requirements and provide optimal health care to a diverse population. Nursing schools thus have an important goal of educating nurses to ensure they are culturally competent. Culturally competent care improves safety and equity for patients. To measure cultural awareness among nursing students in Sweden, the aim of this study was to translate, adapt and test the validity and reliability of the Swedish version of a cultural awareness scale which has not previously been tested.

**Methods:**

A total of 158 nursing students from three universities in Sweden completed the 36-item questionnaire on cultural awareness. Verification of face and content validity and a translation/reverse translation process were first carried out.

**Results:**

The results indicate that one item (no 13) caused weak reliability and validity, and therefore it was removed. The reliability test result (with 35 items) showed Cronbach’s Alpha ranged from 0.60 to 0.87. The Model ChiSq group fit for five factors was 50.44 (31.27–77.06; Df = 5; *p* < 0.001), and the RMSEA was 0.24 (C.I 95 % = 0.18–0.30).

**Conclusion:**

The findings of the validity and reliability tests revealed that the CAS-scale for the 35 items is valid and reliable for use with Swedish nursing students. However, the CAS should be further tested in larger and more diverse samples of nursing students before being used in different socio-cultural settings.

## Background

Due to globalization the world has become more accessible [1], and therefore cultural awareness and cultural competence are becoming important skills for nurses [2]. Nearly eighteen percent of the Swedish population is foreign-born [3], 20.6 % of Canada’s population are immigrants [4] and 28 % of the Australian population are born overseas [5]. Foreign-born residents are shown to have an increased risk of varying health problems such as diabetes [6, 7] malnutrition [8], mental illness [9–13] and psychosomatic problems [6] due to mental stress. This puts demands on nurses to meet the unique needs of people from different cultures. Cultural competence in nursing is about being able to care for patients while taking their cultural background into consideration [2, 14]. In the Swedish Higher Education Act [15] cultural awareness is emphasized and internationalization and raising awareness of different cultural contexts have become essential elements in Swedish higher nursing education. However, previous studies found that there was a lack of cultural competence among nurses, which is a necessary skill in multicultural health care [16–19]. Nursing students who are not trained to be culturally competent often do not correctly address the unique needs and preferences of individuals from cultures different from their own [20]. Thus, it is important to explore whether nursing students in Sweden experience cultural awareness after completing their studies and, depending on the results, continue addressing the four components of cultural competence: cultural awareness, cultural sensitivity, cultural knowledge and cultural skills in nursing education.

To our knowledge there are few previous qualitative studies that have investigated Swedish [16], American (United States) [18] and Canadian nursing students’ [21] cultural awareness. The lack of quantitative studies can be due to the fact that no reliable instrument exists for measuring cultural awareness in the Swedish context among nursing students. Therefore, the aim of this study is to identify and validate an appropriate scale measuring baccalaureate nursing students’ cultural awareness in Sweden.

### Cultural awareness scale

Rew [22] developed a Cultural Awareness Scale (CAS) for nursing students, in English. The scale is based on major cultural competence elements, such as: cultural awareness, cultural sensitivity, cultural knowledge and cultural skills. Cultural awareness is about being aware that a person’s cultural background affects the person’s behaviors and attitudes, and cultural sensitivity is about respecting these cultural differences. Furthermore, developing cultural competence takes time and involves having knowledge of other cultures and learning skills to interact and cooperate with people from varying cultural backgrounds, speaking different languages and having other relationship skills, and this puts demands on behavioral flexibility [22]. The CAS consists of 36 items developed to measure the cultural awareness of students, where cultural awareness is considered to be the minimal level of cultural competence. The scale has been suggested to provide a tangible method for documenting the first stage of the development of cultural competence among nursing students [22]. The scale is based on a Pathways Model analysis [23] and is consistent with the Purnell Model of Cultural Competence theory [24]. The instrument was pilot tested with 72 undergraduate and graduate nursing students in the US (United States). Cronbach’s Alpha was 0.82 for the faculty and 0.91 for students in the overall test. A content validity index of 0.88 was obtained, based on data collected from a seven member expert panel. The CAS validity and reliability was completed by Krainowich-Miller et al. [20] and was found to be comparable to the findings of Rew et al. [22]. The survey utilizes a Likert-type scale ranging from 1 to 7 to indicate how much the students agree or disagree with each statement. 1 = strongly disagree, 4 = no opinion, 7 = strongly agree. The CAS consists of five subscales: general education experience, cognitive awareness, research issues, behaviors/comfort with interaction and patient care/clinical practice.

The Swedish Higher Education Act sets the goals for nursing education (14). One of the main goals is to provide education enabling students to become culturally competent. It is therefore important to discover whether students perceive that they become culturally competent during their nursing studies. As no reliable Swedish instrument was found, the first step was to translate, adapt and test the validity and reliability of the Swedish version of a survey measuring nursing students’ cultural awareness.

## Methods

### Design

This psychometric study is part of larger quantitative cross-sectional study concerning nursing education in Sweden and cultural awareness. Thus, the first part of the study was to translate, adapt and test the validity and reliability of the Swedish version of a survey measuring baccalaureate nursing students’ cultural awareness in the last semester of their nursing education.

Psychometric validation is the process of testing whether the instrument is acceptable in terms of reliability and validity, using the population group for whom the instrument is intended [25]. Different psychometric tests have been utilized in this study to assess face, content and construct validity, and also the reliability of the scale in terms of stability and internal consistency.

Since no appropriate instrument was identified in Sweden the first phase of the research started with identifying an appropriate questionnaire in English, to be translated.

### Instrument

Permission to use the CAS was obtained from Rew et al. [22]. A cover page was added to the questionnaire describing the project, implementation of the study, the ethical considerations and participants’ demographic background.

### Validity test

The process of translation of a scale is usually considered as the first phase of validating it. The scale was translated using a translation-back translation method [26, 27]. The scale was translated first into Swedish and was then translated back into English. The back-translation was compared with the original text to identify any differences in wording, and in essence for face validity. In this regard the purpose of face validity was to ensure the validity of the translation by comparing the Swedish version with English one. In the second step the content validity was carried out to identify if the content of the scale is suitable for the target population [28]. The items and factors were retrieved from an already existing constructed scale for content validity evaluated by a pilot test among students to find out if the content (items) of the translated scale was appropriate for use among Swedish population. For this reason, in the pilot test a margin was added to the right side of the scale to enable students to write comments if they had any difficulties understanding any terms, concepts or questions.

The construct validity utilized a confirmatory factor analysis [28]. The purpose of applying a construct validity test was to investigate whether the scale factors measured what they were supposed to measure, and whether the items significantly correlated to each other. Since, in the original English version of the scale, five factors emerged from the 36 items [22], the confirmatory factor analysis method was selected as an appropriate analysis method for testing the study’s assumptions. The confirmatory factor analysis was performed using a five factor solution method. The Kaiser-Meyer-Olkin (KMO) statistic was used for testing sample adequacy. A KMO of more than 0.60 is considered as an acceptable value [29]. Although there is no universal agreement about sample size for factor analysis, the minimum number should be always be at least 100, or three times the number of items' in a scale (36*3 = 108) [30]. Additionally, Bartlett's test was used to check the assumptions on factorability of the items. If the Bartlett's test does not show a significant level (0.05) it means the variables are not correlated.

The items were grouped into the five pre-determined factors, based on their factor loading. There is no standard cut-off point for factor loading. In general, a factor loading of more than 0.50 is considered as good, but if the purpose of the factor analysis is testing a hypothesis or the sample population is small then a factor loading of 0.30 or even lower may be considered as acceptable [31].

The final model of the scale after factor analysis was to investigate the fitness of the scale with the five factors. A structural equation modeling analysis using Root Mean Square Error of Approximation (RMSEA) was used for this reason. A RMSEA value close to zero indicates a good fit and higher than 0.70 indicates a poor or unacceptable model fit.

### Reliability test

Testing the reliability of the scale was carried out using two methods: the first step was a consistency and a stability test among total of 158 students who participated in the final phase of this study. The consistency of the items within each factor was assessed by Cronbach’s Alpha. The cut-off point for this value was 0.60 which was considered a poor but acceptable value [32–34].

The second step was a split-half analysis was used for testing the stability of the items in the scale using the Guttman split-half coefficient and the Spearman Brown coefficient (cut-off 0.60). Split- half analysis is an alternative method for test-retest analysis, which indicates the stability of the variables over time.

### Procedure

The validity and reliability of the scale were tested among nursing students in their final year of education. A convenience sampling method was used to recruit a sample of nursing students (over 18 years old) in the last semester of their Bachelor of Science nursing education in three different universities in southern Sweden. The convenience sampling method was used for sample recruitment since this study targeted only students in the final year of their education, and a random selection of the participant was not an appropriate option. Data were gathered from three universities in South Sweden that offer nursing programs. Data were collected at one point in the sixth semester (last semester in the undergrad nursing program) during 2014.

To make contact with participants the authors contacted the respective heads of the departments for the undergraduate nursing program by phone or email, to get permission for the study. After the approvals for the study were obtained, the representatives were requested to invite students in the undergraduate nursing program in the sixth semester to participate in an information meeting. One information meeting was held at each university. At the meetings, verbal and written information about the study was provided. After each meeting the CAS-scale questionnaire was given to voluntary participants to fill in during a lecture. Questionnaires were distributed among the students, and they were asked to return them in a sealed envelope that was also provided. The questionnaires were collected after the lecture by the authors.

## Results

A total of 158 nursing students (including 30 who took part in a pilot-test) participated in this study from three universities in southern Sweden. All the participants were undergraduate nursing students in the final semester of their education. The participants were mostly female - 84 % (131), with an age range from 20 to 46 (M = 27.23; SD = 5.6). The number of migrants among the sampled population was 30 (19 %), and 8.9 % (14) of them were born outside Sweden (first generation immigrants), while 10.1 % (16) were born in Sweden but from parents with an immigration background (second generation immigrants).

### Phase 1: Translation validity (face and content validity)

The questionnaire was translated from English into Swedish independently by the two authors with experience in relation to migration and health and quantitative methods. After the independent translations were completed a discussion was held among the research unit until agreement was reached with regard to the translation. A translation-back translation was performed by a professional translator later on to test consistency of the Swedish version against the original text. In the second step a bilingual person who is familiar with the topic refined the translations and assessed equivalence, congruence and any colloquialisms used in the instrument. In the last step, the research unit including three nurses and one sociologist evaluated the whole process along with professional translator, and the Swedish version of the scale was finalized for a pilot test.

A pilot test was carried out with 30 nursing students to investigate the content validity of the scale. We asked students to read and answer all the questions and report any difficulties and problems in the left margin of the questionnaire. The participants found that all 36 items in the questionnaire were clear, concise and easy to understand. The findings only led to minor corrections of wording and layout. These findings indicate that the scale was validated in term of face validity and content, since the Swedish version is faithful to the essence of the original scale and the pilot test and it demonstrated that informants had no difficulty in comprehending the content of the scale.

### Phase 2: Reliability test

The reliability test was conducted using consistency and stability tests on five factors in the questionnaire. The results indicated acceptable and good reliability for factors with regard to: General educational experience (.76), Cognitive awareness (.68), Research issue (.87), and Patient care/clinical practice (.65). The split-half reliability for the above mentioned factors was acceptable as well (please see Table 1). The reliability test also showed, however, that the factor Behaviors/comfort with interaction has a borderline value of 0.49.

**Table 1:** Reliability test for the Swedish version of the cultural awareness scale with five factors

Factors	No.	Cronbach’s Alpha	Guttman split-half coefficient	Spearman Brown coefficient
General educational experiences	14	.76	.65	.66
Cognitive awareness	7	.68	.56	.57
Research issue	4	.87	.86	.86
Behaviors/comfort with interaction	6	.49^a^	.49^a^	.49^a^
Patient care/clinical practice	5	.65	.70	.64

^a^ Low consistency and stability

At this stage another statistical analysis was performed to identify the weakest items within the fourth factor (Behaviors/comfort with interaction). The reliability test was performed one more time with Cronbach’s Alpha “*if item deleted*” method. The result indicated that there is only one item in this factor that causes weak reliability. The statistical analysis suggested removing that weakest item (question number 13: I have noticed that the instructors at this nursing school call on students from minority cultural groups when issues related to their group come up in class) to get an acceptable reliability result. Thus, that item was removed from the scale and the Cronbach’s Alpha reached 0.6, although the Guttman split-half coefficient and Spearman Brown coefficient stayed at the level of 0.50. The stability test of the data using the Spearman Brown coefficient and Guttman split-half coefficient also indicated a relatively poor value, below 0.60.

The inter-correlation between the five factors was tested and the findings indicated a very low correlation between the variables, and in some cases a negative correlation (please see Table 2). The correlation between Cognitive awareness and Behaviors/comfort with interaction is (−.12); and the correlation between Research issue with Patient care/clinical practice is also negative (−.05)

**Table 2:** Inter-factor correlation matrix of the cultural awareness scale

Factors	1	2	3	4	5
1: General educational experiences	**1.00**	.09	.30	.10	.10
2: Cognitive awareness		**1.00**	.01	-.12	.17
3: Research issue			**1.00**	.13	-.05
4: Behaviors/comfort with interaction				**1.00**	.36
5: Patient care/clinical practice					**1.00**

### Phase 3: Validity test (construct validity)

The validity test was performed by Confirmatory Factor Analysis among the five factors. The Kaiser-Meyer-Olkin Measure of Sampling Adequacy (KMO) was 0.63 and Bartlett's Test of Sphericity by Chi square was 1794.26 (DF = 595; Sig = .00). It means that the sample population for the 35 items is mediocre, and acceptable.

The result of factor loading using Confirmatory Factor Analysis (CFA) also indicated that, except for the items 16, 17, and 22, the factor loadings were higher than 0.32. The percentage of variance for the five factors was 44.33. Although the factor loading for three items was low, since they had communalities of more than 0.60, they remained in the final scale. To assess the construct validity of the scale with all five factors that were identified in the original scale and 35 items that were identified using CFA in this study, structural equation modeling analysis was carried out (see Table 3).

**Table 3:** Factor loading of five sub-scales of the cultural awareness scale (35 items variance = 44.33 %)

Item	General educational experiences	Cognitive awareness	Research issue	Behaviors/comfort with interaction	Patient care/clinical practice
1. The instructors at this nursing school adequately address multicultural issues in nursing.	.666	-	-	-	-
2. This nursing school provides opportunities for activities related to multicultural affairs.	.628	-	-	-	-
3. Since entering this nursing school, my understanding of multicultural issues has increased.	.631	-	-	-	-
4. My experiences at this nursing school have helped me become knowledgeable about the health problems associated with various racial and cultural groups.	.653	-	-	-	-
5. I think my beliefs and attitudes are influenced by my culture.	-	.714	-	-	-
6. I think my behaviors are influenced by my culture.	-	.743	-	-	-
7. I often reflect on how culture affects beliefs, attitudes, and behaviors.	-	.504	-	-	-
8. When I have an opportunity to help someone, I offer assistance less frequently to individuals of certain cultural backgrounds.	-	-	-	.569	-
9. I am less patient with individuals of certain cultural backgrounds.	-	-	-	.781	-
10. I feel comfortable working with patients of all ethnic groups.	-	-	-	.523	-
11. I believe nurses’ own cultural beliefs influence their nursing care decisions.	-	.645	-	-	-
12. I typically feel somewhat uncomfortable when I am in the company of people from cultural or ethnic backgrounds different from my own.	-	-	-	.632	-
^a^13. I have noticed that the instructors at this nursing school call on students from minority cultural groups when issues related to their group come up in class.	-	-	-	-	-
14. During group discussions or exercises, I have noticed the nursing instructors make efforts to ensure no student is excluded.	.327	-	-	-	-
15. I think students’ cultural values influence their classroom behaviors (e.g. asking questions, participating in groups, offering comments).	-	.703	-	-	-
16. In my nursing classes, my instructors have engaged in behaviors that may have made students from certain cultural backgrounds feel excluded.	.254	-	-	-	-
17. I think it is the nursing instructor’s responsibility to accommodate students’ diverse learning needs.	-	.206	-	-	-
18. My instructors at this nursing school seem comfortable discussing cultural issues in the classroom.	.684	-	-	-	-
19. My nursing instructors seem interested in learning how their classroom behaviors may discourage students from certain cultural or ethnic groups.	.491	-	-	-	-
20. I think the cultural values of the nursing instructors influence their behaviors in the clinical setting.	-	.546	-	-	-
21. I believe the classroom experiences at this nursing school help students become more comfortable interacting with people from different cultures.	.483	-	-	-	-
22. I believe some aspects of the classroom environment at this nursing school may alienate students from some cultural backgrounds.	.201	-	-	-	-
23. I feel comfortable discussing cultural issues in the classroom.	-	-	-	-	.507
24. My clinical courses at this nursing school have helped me become more comfortable interacting with people from different cultures.	.321	-	-	-	-
25. I feel that the instructors at this nursing school respect differences in individuals from diverse cultural backgrounds.	.351	-	-	-	-
26. The instructors at this nursing school model behaviors that are sensitive to multicultural issues.	.717	-	-	-	-
27. The instructors at this nursing school use examples and/or case studies that incorporate information from various cultural and ethnic groups.	.502	-	-	-	-
28. The faculty at this nursing school conducts research that considers multicultural aspects of health-related issues.	-	-	.782	-	-
29. The students at this nursing school have completed theses and dissertation studies that considered cultural differences related to health issues.	-	-	.829	-	-
30. The researchers at this nursing school consider the relevance of data collection measures for the cultural groups they are studying.	-	-	.892	-	-
31. The researchers at this nursing school consider cultural issues when interpreting findings in their studies.	-	-	.884	-	-
32. I respect the decisions of my patients when they are influenced by their culture, even if I disagree.	-	-	-	-	.625
33. If I need more information about a patient’s culture, I would use resources available onsite	-	-	-	-	.578
34. If I need more information about a patient’s culture, I would feel comfortable asking people I work with.	-	-	-	-	.810
35. If I need more information about a patient’s culture, I would feel comfortable asking the patient or family member.	-	-	-	-	.825
36. I feel somewhat uncomfortable working with the families of patients from cultural backgrounds different than my own.	-	-	-	.647	-
Percentage of variance	12.666	9.344	8.634	7.950	5.740

^a^Removed from the validity test after reliability test

The result of structural equation modeling analysis indicated that the model of having five factors (with 35 items) within the CAS is perfectly adequate. The Model Chi Sq group fit for five factors was 50.44 (31.27–77.06; Df = 5; *p* < 0.001), and the RMSEA was 0.24 (C.I 95 % = 0.18–0.30).

## Discussion

The results indicate that the original subscales are suitable for the Swedish version; however, one item was removed due to the lack of validity and reliability. The Swedish version of the scale has, in total, 35 items grouped into five factors, instead of 36. It is difficult to obtain reliable items in a scale when the research deals with sensitive and complex subjects, which is the case in our study. It should be noted that this scale has been validated for quite a small group of nursing students in their final semester in southern Sweden [35]. However, the study results are mostly consistent with the findings of previous studies [20, 22].

The findings also revealed that the reliability test result is low for three of the sub-scales This finding may be due to the small sample population or small number of items, which leads to a low reliability test result [36]. Therefore, we recommend further testing of validity in a larger group with a larger sample to use the scale in a diverse population. Although the results indicated high stability of all five subscales, the subscales are not in direct positive correlation with each other. Thus, having a total score for the whole scale is not recommended. In the original English CAS scale, two subscales of “general educational experience” and the “cognitive awareness subscale” are also negatively correlated with “behaviors/comfort with interactions” [22]. However, our findings indicate that “cognitive awareness” is also negatively correlated with “behaviors/comfort with interactions”, and “research issue” correlated negatively with “patient care/clinical practice”. This result is not in line with the results of earlier studies by Rew et al. [22] and Krainovich-Miller et al. [20] in this regard. This finding is also in contrast with the findings of the study by Rew et al. [37] that comprised factors “cognitive awareness” with “behaviors/comfort with interaction”. Having a negative correlation between factors which are identified in our study suggests that each factor should stand by itself and cannot be combined with other factors. It also means that having a total score for 35 items for assessing general cultural awareness would not be an option in this scale. The differences between our study findings and those of the earlier study by Rew et al. [37] can be explained by the possible diversity in the sample population, educational system and educational content. The studies by Rew et al. [22] and Krainovich-Miller et al. [20] included data from a university with BS, MS and PhD students, and with more foreign-born participants. However, our sample population was not as diverse and mostly represented the general Swedish population, with 20 % of the participants having a foreign background, and it also contained only BS students in their final year of education. Furthermore, our study comprised BS students in their final year of education that may not have been as engaged in research practice as MS and PhD students. Thus, the negative correlation between “research issue” and “patient care/clinical issue” may arise from sample population differences in terms of having only undergraduate students in this study.

Another explanation may be the differences in the educational system, and educational content. This scale was tested in the Swedish nursing education program by three different universities in Southern Sweden. In Sweden, at the national level, nursing students receive both professional and academic degrees at the end of their nursing education [15]. Therefore, the sample population of this study includes students in the last semester in the BS program to ensure that students were at same level at their education and that they take part in the whole training, which covers both clinical experience and academic training. Furthermore, the Swedish Higher Education Authority at the national level is responsible for the quality evaluation, legal oversight and statistical follow- up of Swedish Universities. This means that the students who participated in this study were from three different universities but they should have the same national educational goals. The Swedish Higher Education ACT [15] highlights the importance of cultural competence for nurses. However, previous studies indicated that there is still a lack of shared understanding about the concept of cultural competence among health care providers and health educators in the Swedish [14], Australian [17], US [18, 19] and Canadian contexts [21].

## Conclusion

The findings of this study support the reliability of the CAS using 35 items within the previous studies by Rew et al. [22] and Krainovich-Miller et al. [20]. Although the result indicates that the CAS scale is validated and is a reliable scale for use among nursing students in a Swedish context, it should be noted that the scale was tested among a small sample population (BS level) at three universities. Thus, future use of this scale among postgraduate students is not recommended without additional validity testing. We also recommend that further studies with a larger sample population are carried out to compare the cultural awareness of nursing students based on the students’ cultural/ethnic backgrounds. Furthermore, we also agree with Krainovich-Miller et al. [20] regarding the need to refine the scale before applying it to a different socio-cultural setting.

## Ethics and consent to participate

All participants received verbal and written informed consent that they could withdraw from the study at any time with no further explanation required. They were informed about the aims of the study, focusing on cultural awareness from their perspective, about the implementation of the study in accordance with Swedish law concerning regulation of ethical research involving humans [38] and common ethical principles definite in Declaration of Helsinki [39]. The questionnaires were anonymous and coded by number to preserve the confidentiality of the participants’ data. The analysis and presentation of the data was done at a group level and in a way that concealed the participants’ identity. All the collected data was stored in a locked space which could only be accessed by the authors [39]. The present research study posed no physical or mental risk to the participants and did not treat participants’ personal data and therefore, according to Swedish Law [38] approval by an official research committee was not required [38, 39].

## Consent to publish

Not applicable

## Availability of data and materials

All the data supporting the findings is contained within the manuscript.

## Abbreviations

BS: Bachelor of Science
CAS: Cultural Awareness Scale
CFA: confirmatory factor analysis
CI: confidence interval
Cut-off: Spearman Brown coefficient
DF: degree of freedom
KMO: Kaiser-Meyer-Olkin
M: median
MS: Master of Science
No: number
PhD: Doctor of Philosophy
RMSEA: Root Mean Square Error of Approximation
SD: standard deviation
Sig: significant
US: United States

## References

[1] Future capacity needs in managing the health Aspects of migration. [http://publications.iom.int/system/files/pdf/wmr2010_capacity_needs_health_spects.pdf]. Accessed 7 Apr 2016.

[2] Leininger MM, McFarland MR (2006). Culture care diversity and universality : a worldwide nursing theory, 2 edn. Sudbury, Mass.

[3] Statistiska centralbyrån (2010). Tabeller över Sveriges befolkning 2009 = [Tables on the population in Sweden 2009].

[4] Immigration and Ethnocultural Diversity in Canada. [http://www12.statcan.gc.ca/nhs-enm/2011/as-sa/99-010-x/99-010-x2011001-eng.pdf]. Accessed 7 Apr 2016.

[5] Australian Bureau of Statistics. [http://www.abs.gov.au/ausstats/abs@.nsf/mf/3412.0/]. Accessed 7 Apr 2016.

[6] Toselli S, Gualdi-Russo E, Marzouk D, Sundquist J, Sundquist K (2014). Psychosocial health among immigrants in central and southern Europe. Eur J Public Health.

[7] Mezuk B, Cederin K, Li X, Rice K, Kendler KS, Sundquist J, Sundquist K (2014). Immigrant enclaves and risk of diabetes: a prospective study. BMC Public Health.

[8] Global Nutrition report, ACTIONS AND ACCOUNTABILITY TO ACCELERATE THE WORLD’S PROGRESS ON NUTRITION. [http://www.unscn.org/en/home/]. Accessed 7 Apr 2016.

[9] Ekblad S, Marttila A, Emilsson M (2000). Cultural challenges in end-of-life care: reflections from focus groups' interviews with hospice staff in Stockholm. J Adv Nurs.

[10] Bäärnhielm S (2003). Clinical encounters with different illness realities : Qualitative studies of illness and restructuring of illness meaning among two cultural groups of female patients in a multicultural area of Stockholm.

[11] Zolkowska K, Cantor-Graae E, McNeil TF (2001). Increased rates of psychosis among immigrants to Sweden: is migration a risk factor for psychosis?. Psychol Med.

[12] Gilliver SC, Sundquist J, Li X, Sundquist K (2014). Recent research on the mental health of immigrants to Sweden: a literature review. Eur J Public Health.

[13] Khan A, Leventhal RM, Khan S, Brown WA (2002). Suicide risk in patients with anxiety disorders: a meta-analysis of the FDA database. J Affect Disord.

[14] Jirwe M (2008). Cultural Competence in Nursing Department of Neurobiology, Care Sciences and Society, Division of Nursing.

[15] SFS, Socialdepartementet (1992). Högskolelag (Higher Education Law).

[16] Momeni P, Jirwe M, Emami A (2008). Enabling nursing students to become culturally competent--a documentary analysis of curricula in all Swedish nursing programs. Scand J Caring Sci.

[17] Northam HL, Hercelinskyj G, Grealish L, Mak AS (2015). Developing graduate student competency in providing culturally sensitive end of life care in critical care environments - A pilot study of a teaching innovation. Aust Crit Care.

[18] Sargent SE, Sedlak CA, Martsolf DS (2005). Cultural competence among nursing students and faculty. Nurse Educ Today.

[19] Mareno N, Hart PL (2014). Cultural competency among nurses with undergraduate and graduate degrees: implications for nursing education. Nurs Educ Perspect.

[20] Krainovich-Miller B, Yost JM, Norman RG, Auerhahn C, Dobal M, Rosedale M, Lowry M, Moffa C (2008). Measuring cultural awareness of nursing students: a first step toward cultural competency. J. Transcult. Nurs/Transcultural Nursing Society.

[21] Vandenberg H, Kalischuk RG (2014). Conceptualizations of culture and cultural care among undergraduate nursing students: an exploration and critique of cultural education. J Cult Divers.

[22] Rew L, Becker H, Cookston J, Khosropour S, Martinez S (2003). Measuring cultural awareness in nursing students. J Nurs Educ.

[23] Rew L (1996). Affirming cultural diversity: a Pathways model for nursing faculty. J Nurs Educ.

[24] Purnell LD, Paulanka BJ (2008). Transcultural health care : a culturally competent approach.

[25] Bowling A (2009). Research methods in health : investigating health and health services.

[26] Behling O, Law KS (2000). Translating questionnaires and other research instruments: problems and solutions.

[27] Rode N (2005). Translation of measurement instruments and their reliability: An example of job-related affective well-being scale. Metodoloski zvezki.

[28] Jordan C, Hoefer RA, Thyer BA (2001). Reliability and validity in quantitative measurement. Social work research methods edn.

[29] Brace N, Kemp R, Sneglar R (2006). SPSS for Psychologists.

[30] Mundfrom DJ, Shaw DG, Ke TL (2005). Minimum Sample Size Recommendations for Conducting Factor Analyses. Int J Test.

[31] Comrey AL, Lee HB (1992). A first course in factor analysis.

[32] Breznau N (2010). Valerie Møller, Denis Huschka and Alex C. Michalos (Eds.) (2008). Barometers of quality of life around the globe: How are we doing? Social Indicators Research Series. IJPOR.

[33] Altman DG. Inter-rater agreement. Practical statistics for medical research*.* 1991;5:403–49.

[34] Kehoe J (1995). Basic item analysis for multiple-choice tests.

[35] Spector P (1992). Summated rating scale construction: an introduction.

[36] McCrae RR, Kurtz JE, Yamagata S, Terracciano A (2011). Internal consistency, retest reliability, and their implications for personality scale validity. Pers Soc Psychol Rev.

[37] Rew L, Becker H, Chontichachalalauk J, Lee HY (2014). Cultural diversity among nursing students: reanalysis of the cultural awareness scale. J Nurs Educ.

[38] Förordning om etikprövning av forskning som avser människor (Swedish law: Regulation of ethical research involving humans). [http://www.riksdagen.se/webbnav/index.aspx?nid=3911&bet=2003:460 (accessed 15 March 2010).]

[39] World Health Organization (2008). United Nations. Office of the High Commissioner for Human Rights.

